# CD38 Expression by Circulating and Skin-Infiltrating Lymphocytes from Sezary Syndrome Patients: A Flow Cytometry and Immunohistochemistry Study

**DOI:** 10.1155/2022/3424413

**Published:** 2022-02-24

**Authors:** Pietro Quaglino, Mauro Novelli, Paolo Fava, Erika Ortolan, Chiara Astrua, Luca Tonella, Carlo Francesco Tomasini, Rebecca Senetta, Simone Ribero, Renata Ponti, Maria Teresa Fierro, Ada Funaro

**Affiliations:** ^1^Dermatologic Clinic, Department of Medical Sciences, University of Turin Medical School, Torino, Italy; ^2^Laboratory of Immunogenetics, Department of Medical Sciences, University of Torino, 10126 Torino, Italy; ^3^IRCCS Fondazione Policlinico San Matteo, Department of Medical-Surgical, Diagnostic and Pediatric Sciences, Dermatologic Clinic, University of Pavia School of Medicine, Pavia, Italy; ^4^Department of Oncology, Pathology Unit, University of Turin, 10126 Turin, Italy

## Abstract

**Background:**

Reports on the expression of CD38 in Sézary syndrome (SS), erythrodermic primary cutaneous T cell lymphoma with leukemic involvement, are limited. The aim of the present study is the analysis of the expression of CD38 by skin-infiltrating mononuclear cells and circulating T lymphocytes in a cohort of SS patients.

**Methods:**

SS patients diagnosed since 1985 in our clinic were retrospectively analyzed for CD38 expression in biopsy and blood samples by immunohistochemistry and flow cytometry, respectively.

**Results:**

SS patients show a predominant CD38-negative phenotype on both skin and blood. A subgroup of patients was found expressing CD38 (12 cases) in either the skin (>25% cell infiltrate) or blood (CD4+CD38+ >50%), among whom 4 in the blood, 7 in the skin, and 1 in both blood and skin.

**Conclusion:**

The implications of these observations may be twofold: the relevance in basic science is related to a potential role in immune defense regulation, whilst in perspective CD38 may become a target for antibody therapy, considering the availability of different anti-CD38 monoclonal antibodies.

## 1. Background

Sézary syndrome (SS), the erythrodermic and leukemic variant in the spectrum of cutaneous T cell lymphomas (CTCL), is characterized by a clinical triad including erythroderma, enlarged peripheral nodes, and atypical circulating T lymphocytes with “cerebriform” nuclei [1]. Sézary cells show a characteristic “central memory” CD4+ phenotype, with lack of CD26 and CD7 [2–6] and expression of “central memory” markers, CCR4 and KIR3DL2 (CD158k molecule) [2–6]. A recent consensus by the European Organization for Research and Treatment of Cancer (EORTC) suggested to define blood involvement (B2 according to the TNMB classification) [1] on the basis of phenotypical criteria in the presence of more than 1,000/mm^3^ CD4+CD26- or CD4+CD7- cells [7].

CD38 is a type II transmembrane glycoprotein expressed by different cells without lineage restriction [8]. The function of the CD38 molecule is pleiotropic, initially defined as receptor able to transduce activation signals; it was then considered as an adhesion molecule with CD31 (PECAM-1) as counterreceptor. Another function of CD38 is to act as an ectoenzyme, able to metabolize extracellular nicotinamide adenine dinucleotide (NAD+) and to produce adenosine diphosphate ribose (ADPR) and cyclic adenosine diphosphate ribose (cADPR), both cytoplasmic second messengers. Through the generation of ADPR, CD38 takes part in the production of extracellular adenosine (ADO), a potent tolerogenic compound acting as potent immune suppressor [8].

Reports on the CD38 expression by circulating or skin-infiltrating lymphoid cells in SS are very limited. In our previous report [2], CD38 expression on peripheral blood lymphocytes (PBL) from SS patients was significantly lower than in healthy subjects.

## 2. Question Addressed

The aim of the present study is to analyze the expression of CD38 in skin-infiltrating mononuclear cells and circulating T lymphocytes from SS patients.

The relevance of the analysis of CD38 in SS patients is twofold. The expression of CD38 is associated with the production of the immune-suppressor molecule adenosine, which could therefore represent one of the mediators of immune downregulation in these patients [8]. Moreover, it is well known that SS cells lack CD26 expression [2–6]. CD26, a transmembrane glycoprotein with dipeptidyl peptidase IV (DPPIV/CD26) activity, represents the main cellular binding protein for adenosine deaminase (ADA), the enzyme involved in adenosine degradation [9]. The absence of CD26 may determine a reduced degradation of adenosine with upregulation of immune-suppressive signals.

The expression of CD38 could be also relevant as a potential therapeutic target (anti-CD38 antibodies daratumumab and isatuximab approved in the US, Europe, and Italy for the treatment of multiple myeloma) [10, 11].

## 3. Materials and Methods

### 3.1. Patients

Seventy-six SS patients were extracted from the Cutaneous Lymphoma Database of the Dermatologic Clinic, University of Turin, which includes all the cases of primary cutaneous lymphoma diagnosed since 1985.

SS diagnosis was established based on the criteria available at the time of each patient presentation using a combination of clinical, histologic, and immunohistochemical findings; all the cases were reclassified according to the WHO/EORTC classification [1, 6]. Inclusion criteria for this retrospective study were represented by the availability of the analysis of CD38 expression at diagnosis either in the blood and/or in the skin and at least 30% of circulating CD4+CD26- cells, to avoid potential biases associated with the presence of limited blood involvement [12].

The study was performed according to the principles of good clinical practices and the Declaration of Helsinki principles. Study approval was obtained from the local ethical committee of the “Azienda Ospedaliera Città della Salute e della Scienza di Torino, Italy.”

### 3.2. Immunohistochemistry

Immunohistochemistry on frozen sections was performed by the standard streptavidin-biotin-peroxidase method (LSAB2 plus kit; Dako, Glostrup, Denmark) using a wide panel of monoclonal antibodies. CD38 expression was detected using the Leu-17 clone (BD Biosciences Europe). CD38 expression in biopsy samples taken at diagnosis was available in 63 out of 76 patients. All samples were evaluated by two independent pathologists and scored as follows: CD38 expression in ≤5% of the lymphocyte infiltrate = negative (-); in >5-10% = almost negative (+/-); in >10%-25% = weakly positive (+); in >25% = positive (++).

### 3.3. Flow Cytometry

Our archives contain flow cytometry standard files collected since 1985 using FACSCan (software, CONSORT 30 and FACSCan Research), FACSCalibur (software, CellQuest), since 2009, FACSCanto II cytometer (BD Biosciences, San Jose, CA), and more recently since march 2015, using Navios Cytometer (Coulter-Beckman) equipped with three lasers: blue (488 nm), red (638 nm), and violet (405 nm) and Kaluza software for analysis (Beckman Coulter).

All 76 patients had blood sample available at diagnosis. A total of 87 blood samples taken from age- and sex-matched healthy donors were included as normal controls.

The procedures for flow cytometry tests and analyses have been previously reported [2]. For CD38 expression, anti-CD38-PE or PE-Cy7 (clone HB-7) was purchased by BD Biosciences, San Josè, CA, USA. Samples were considered CD38-positive for this study purposes if the CD4+CD38+ cell subset was >50% of total circulating lymphocytes, according to the maximum percentage of CD4+CD38-positive cells from healthy donors (48%) and to literature data [13].

To investigate simultaneously the CD38 expression in the CD4+CD26- circulating subset, blood samples from 4 SS patients in follow-up were stained with a combination of anti-CD4-FITC (clone MT-130, Dako), anti CD26-PE (clone L272, BD), and anti-CD38-APC (clone IB4, Aczon) for 30 min at 4°C. Then, erythrocytes were lysed using a standard procedure and samples were acquired and analyzed by a FACSCanto 1 and Diva software. In 3 of these patients, PD-1 expression (CD279/MIH4 clone, PE conjugated, BD Pharmingen) was evaluated on the CD4+ subset.

### 3.4. Statistical Analysis

The results are presented as median and 25th and 75th percentile. The Mann–Whitney *U* test and the Kruskal–Wallis with Dunn post hoc test were used to compare data, giving similar results. *P* < 0.05 was considered statistically significant.

## 4. Results

### 4.1. Patient Characteristics

This study included 76 patients for whom CD38 expression on circulating cells was available. For 63 of these patients, CD38 expression on a skin biopsy was also available. The whole SS patient cohort included 39 males and 37 females, with a median age at diagnosis of 64 years (range: 33-102). Median CD3+CD4+/CD3+CD8+ ratio at diagnosis was 14.5 (range: 1.5-98); 30 (39.4%) patients had a CD3 + CD4 + /CD3 + CD8 + ratio < 10 at diagnosis.

The percentage of circulating CD4+CD26- subset ranged between 30% and 97%. The CD4+CD7- subset in the same patients ranged between 1% and 98% with 32 cases showing less than 40% CD4+CD7- cells. All patients had >1,000/mm^3^ CD4+CD26- cells, whilst 25 (32.9%) had less than 1,000/mm^3^ CD4+CD7- cells.

### 4.2. CD38 Expression in Skin Biopsies

**Figure 1:**
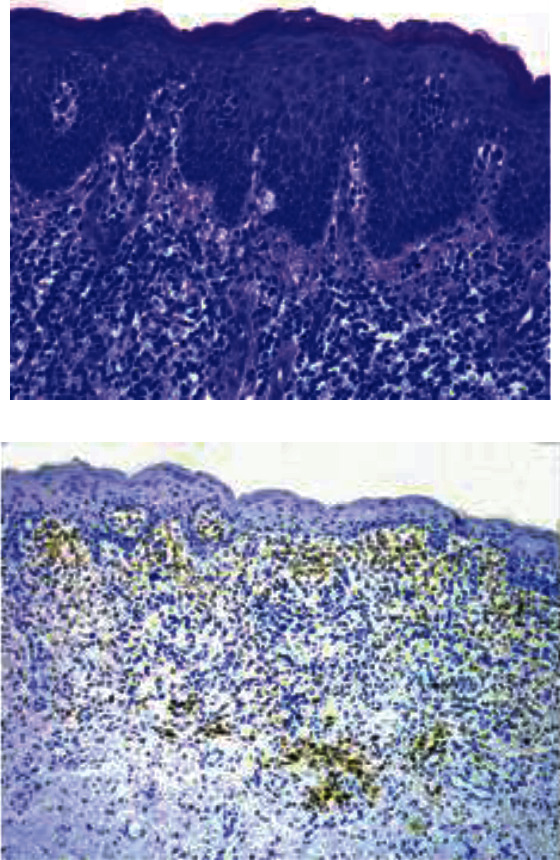
(a) Histology and (b) immunohistochemistry of a representative SS patient. (a) Hematoxylin-eosin staining shows a diffuse superficial tumor infiltrate with epidermotropism (magnification = 10x). (b) Immunohistochemistry shows CD38 expression (>25% cell infiltrate) particularly in the upper dermis superficial infiltrate at the dermal epidermal junction and in scattered large cells in the deep dermis.

In 17 out of 63 skin samples available for analysis, CD38 was expressed by less than 5% of infiltrating lymphocytes, in 22 samples CD38 was expressed by 5% to 10%, in 16 by 10% to 25%, and in 8 by more than 25% of infiltrating lymphocytes. Therefore, in most patients (39/63; 62%), CD38 was absent or expressed in less than 10% of the lymphocyte infiltrates. Only 8 patients (10.5%) had a significant amount of CD38+ cells (>25%) in the context of the cell infiltrate (Figure 1; Table 1).

### 4.3. CD38 Expression in Blood Samples

**Figure 2:**
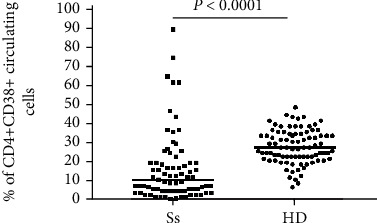
Flow cytometry data showing the percentage of CD4+CD38+ lymphocytes in the peripheral blood from SS patients at diagnosis and healthy donors (HD). Each dot represents a single individual. The horizontal bar represents the median value.

**Figure 3:**
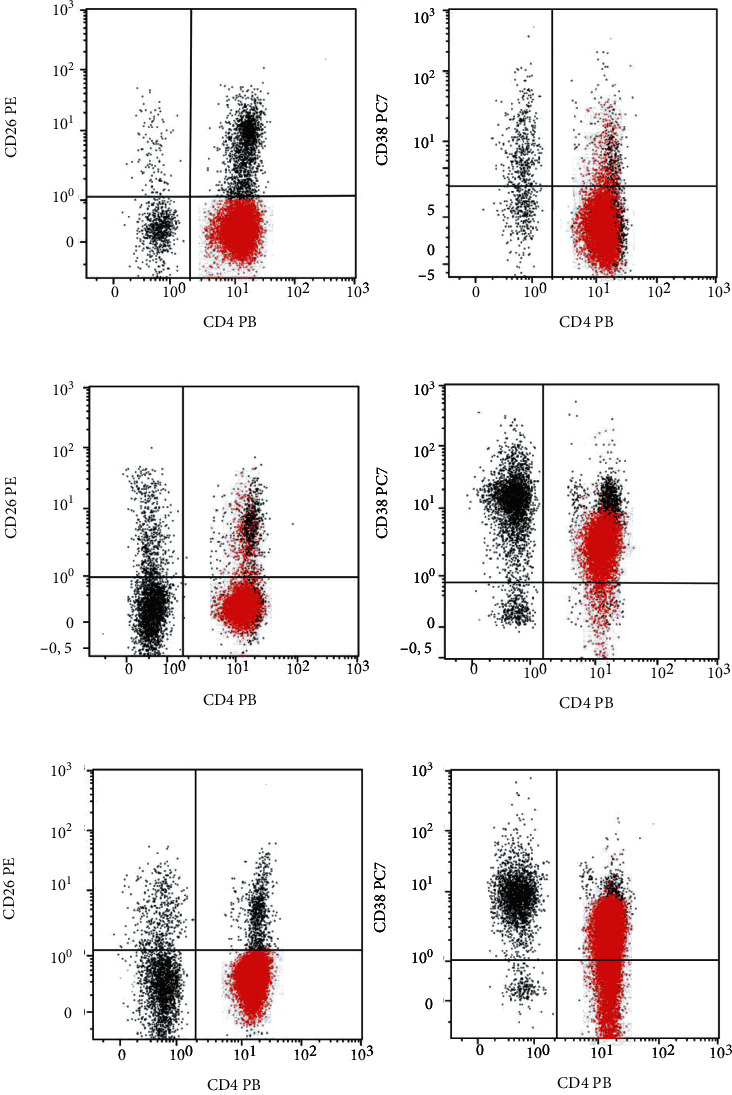
Flow cytometry dot plot of representative cases of CD38+ expression on circulating CD4+CD26- neoplastic T cells. (a): negative CD38 expression; (b) positive CD38 expression; (c): CD38 expression in a fraction of CD4+CD26- cells.

The percentage of the circulating CD4+CD38+ subset in SS patients was significantly lower as compared with that of the age- and sex-matched healthy donors (median in SS = 9%; 25th–75th percentiles = 4%–19% versus median in healthy controls = 27%; 25th–75th percentiles = 22%–34%) (Mann–Whitney test: *P* < 0.0001) (Figure 2). However, as shown in Figures 2 and 3, a subgroup of SS patients displayed high percentage of CD4+CD38+ cells. Indeed, 5 out of 76 patients (6.6%) had more than 50% CD4+CD38+ circulating cells (Table 1). In 3 of these patients, the percentage of CD4+CD26- and CD4+CD38+ cells was similar (89% vs. 89%; 64% vs. 58%; 74% vs. 81%), whilst in one the percentage of CD4+CD38+ cells was slightly lower than that of CD4+CD26- cells (61% vs. 89%). Finally, in one patient, the percentage of CD4+CD38+ cells was remarkably higher than that of CD4+CD26- cells (61% vs. 36%). Only one patient showing high CD38 expression in CD4+ blood cells had >25% CD38-positive cells also in the skin lymphocyte infiltrate.

To compare CD38 expression levels in patients and healthy controls, the expression of CD38 was measured as median fluorescence intensity (MFI) in CD4+CD26-CD38+ cells from 13 SS patients enrolled from 2015 onwards and 23 healthy subjects. The MFI of CD38 was slightly lower in 12 out of 13 patients as compared with that of CD4+CD26- cells from healthy subjects (Figure 1 suppl).

The subpopulation of CD4+CD26-CD38+ cells was further analyzed by multiparametric flow cytometry in 4 SS patients during follow-up (Figure 2 suppl). All these patients had more than 90% CD4+CD26- circulating lymphocytes: two had less than 5% CD4+CD26-CD38+ cells, whilst the other two had 10.8% and 16% CD4+CD26-CD38+ cells, respectively, confirming the expression of CD38 in a fraction of neoplastic cells. PD-1 expression was constantly detected in CD4-positive Sézary cells in the same patients (Figure 3 suppl).

No differences in clinical features were found between the two groups according to age and gender: patients with CD38+ cells tended to show higher numbers of circulating Sézary cells as well as CD4/CD8 ratios, even if this difference was not statistically significant. No difference in overall survival was found between the two groups (median overall survival: 22.2 months, range 1.6 months–9 years in the CD38+ group; 25.2 months, range 2.9 months–12 years in the CD38- patients) (Table 1 Supplementary).

## 5. Discussion

This paper reports the results of a retrospective analysis of CD38 expression in skin and blood samples from a series of 76 SS patients diagnosed, treated, and followed up at the Dermatologic Clinic of the University of Turin since 1985.

Our results confirmed that CD38 is not generally expressed by lymphoid cells in this group of patients, as reported in our previous study [2]. Moreover, a decreased expression of this marker was clearly confirmed by the significantly lower MFI of CD38 on the CD4+CD26- cells in SS patients with respect to HD. This observation is in keeping with the results recently shown also reported by Najidh et al. in 16 SS patients [6].

However, a new finding of the present work is that a small group of SS patients may express CD38, in both skin and blood lymphocytes at diagnosis (Table 1). Indeed, 12 patients expressed CD38 in more than 50% of CD4+ circulating lymphoid cells and/or 25% of skin infiltrates, among whom 4 in the blood, 7 in the skin, and 1 in both blood and skin. However, as the multiparametric flow cytometry evaluations of CD4+CD26-CD38+ cells were not routinely performed in these patients at the time of enrollment, we are aware that a major limitation of this study is the lack of complete data concerning CD38 expression by neoplastic CD4+CD26- or TCR Vbeta-positive cells. The literature reports limited amount of data. A CD38+ phenotype was described in a single case who remarkably had no skin lesions with blood and bone marrow infiltration by atypical cells with ultrastructural features resembling Sézary cells lacking most T lymphoid markers (CD2, CD4, CD3, CD5, and CD8) [14]. In a small series of MF patients, CD38 expression was suggested to correlate with the stage, being higher in the skin infiltrate of patients with tumor stage with respect to that found in patients with patch/plaque stage disease [15]. Torrealba et al. found an increase in the levels of soluble CD38 in SS patients [16].

The implications of the conclusions inferred from the results of this study are twofold. Firstly, from a biologic point of view, special attention deserves the relationship between CD38 and CD26 and immunity. The expression of CD38 (potentially involved in increased adenosine production) coupled with the lack of CD26 (leading to reduced adenosine degradation) [9] could generate an imbalance between the generation and degradation of adenosine contributing to marked local extracellular ADO increase thus switching the microenvironment towards immunosuppression. ADO is an immunosuppressive molecule with multiple functions [17], which is generated through the concerted action of surface molecules endowed with enzymatic functions. Two adenosinergic pathways exist: the canonical pathway is started by nucleoside-triphosphate diphosphohydrolase/CD39 that converts ATP to AMP. AMP is then fully converted to ADO by ecto-5′-nucleotidase/CD73 [18], which mediates its immunoregulatory functions by binding to one of four adenosine receptors (AR). The noncanonical pathway is started by nicotinamide adenine dinucleotide (NAD+)-glycohydrolase/CD38 that converts NAD+ to ADPR. ADPR is then converted by CD203a (PC-1) to AMP that is subsequently metabolized to ADO by CD73 [19]. Based on the results of this study, it is tempting to speculate that in a subgroup of SS patients CD38 could mediate ADO production by the noncanonical pathways thus contributing to local immunosuppression.

SS patients are characterized by a severe impairment of the immune system, which is intrinsic to the disease, is worsened by chemotherapeutic treatments, and gives rise to a high incidence of infections [1]. In this scenario, the high expression of PD-1 by neoplastic cells which is an established feature of this disease [3] also found in our study could represent a further mechanism contributing to the generation of an immune-suppressive microenvironment. The PD-1/PD-L1 axis is associated with the upregulation of an immune-suppressive T cell response; hence, its inhibition is currently being explored to enhance antitumor T cell immunity with certain successful outcomes. However, PD-1 has also emerged as a central tumor suppressor in T cell lymphomas [20], suggesting that checkpoint inhibitors might have the potential to accelerate expansion of tumoral T cell clones. Similarly, we cannot exclude that an adenosine-rich milieu—due to CD38 expression in a relevant fraction of neoplastic cells—exerts its inhibitory effects against SS themselves in an autocrine fashion. This aspect requires careful evaluation in order to understand the clinical significance of CD38 in SS, although increasing evidence indicates that, regardless of the tumor cell type expressing CD38, its effect is immunosuppressive and tumor-promoting [21].

The second point of interest is related to CD38 expression by SS as potential therapeutic target in a subgroup of patients. Today, the armamentarium of anti-CD38 antibodies includes daratumumab and recently isatuximab, both FDA approved in myeloma therapy [9, 10]. It is tempting to envision that the blockade of the CD38 enzymatic pathway could exert a positive impact on the immune regulation processes and promote the restoring of the immune response.

The results of the present study should be confirmed in larger patient cohorts studying CD38 expression in the neoplastic clonal cells characterized by the lack of CD7/CD26 and expression of a single Vbeta chain. Another important issue to address in the future is the detailed characterization of the phenotypical features of the CD38-positive subgroup of patients and the definition of the potential biological significance of CD38 expression in the production of ADO. Overall, these preliminary data could represent the starting point for the development of clinical trials using anti-CD38 antibodies alone or in combination with anti-PD-1 for the treatment of the small subgroup of SS patients who express CD38 and would likely benefit from such targeted therapy.

## Figures and Tables

**Table 1:** Immunohistochemistry and flow cytometry analysis in 12 patients with high CD38 expression in either the blood (>50%) or the skin (>25%).

Patient	Sex/age	Skin	Blood (%)		
		CD38^§^	CD4+CD38+	CD4+CD26-	CD4+
SS-1	F/47	++	61	89	93
SS-2	F/76	++	35	58	78
SS-3	F/83	++	25	53	67
SS-4	M/84	++	19	91	94
SS-5	M/51	+	61	36	63
SS-6	M/47	+	89	89	94
SS-7	F/54	+	64	58	73
SS-8	F/88	NA	74	81	96
SS-9	M/62	++	12	52	82
SS-10	F/62	++	4	80	92
SS-11	F/49	++	3	84	91
SS-12	M/79	++	25	51	80

^§^CD38 expression in the skin: + = 10%–25%; ++ = >25%.

## Data Availability

The Excel data used to support the findings of this study are available from the corresponding author upon request.

## Supplementary Materials

Supplementary 1 Figure 1 suppl: CD38 median fluorescence intensity (MFI) in CD4+CD26- cells from 13 SS patients enrolled from 2015 onwards, compared with 23 HD. Figure 2 suppl: coexpression of CD38 and CD26 in CD4+ cells. Multiparametric flow cytometry analysis of CD38 expression in peripheral blood lymphocytes from 4 patients with SS at follow-up. Plots show the immune profile of CD4+ cells. The table summarizes the percentage of the indicated cell subpopulation in each patient. Figure 3 suppl: coexpression of PD-1 and CD38 in CD4+ cells.

Supplementary 2 Table 1 Supplementary: clinical characteristics of CD38+ and CD38- patients.
