# Supplementary material for: CD38 Expression by Circulating and Skin-Infiltrating Lymphocytes from Sezary Syndrome Patients: A Flow Cytometry and Immunohistochemistry Study
Source: Dis Markers. 2022 Feb 24;2022:3424413. doi: 10.1155/2022/3424413 (PMC8896155; doi:10.1155/2022/3424413)
Supplement: Supplementary 1 — Figure 1 suppl: CD38 median fluorescence intensity (MFI) in CD4+CD26- cells from 13 SS patients enrolled from 2015 onwards, compared with 23 HD. Figure 2 suppl: coexpression of CD38 and CD26 in CD4+ cells. Multiparametric flow cytometry analysis of CD38 expression in peripheral blood lymphocytes from 4 patients with SS at follow-up. Plots show the immune profile of CD4+ cells. The table summarizes the percentage of the indicated cell subpopulation in each patient. Figure 3 suppl: coexpression of PD-1 and CD38 in CD4+ cells. [file 3424413.f1.pdf]

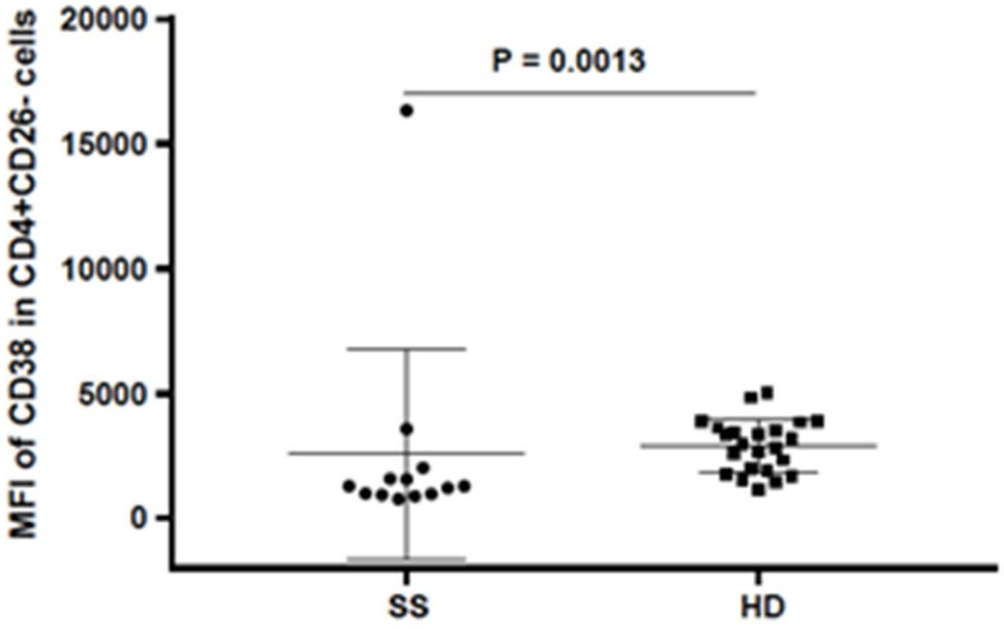

Figure 1 Suppl

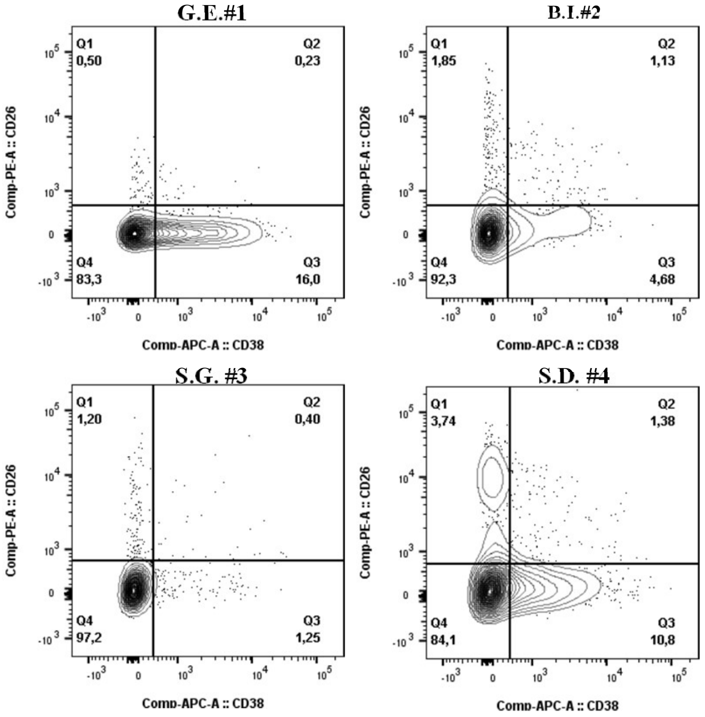

Patients	CD3+CD4+ (%)	CD4+CD26- (%)	CD4+CD26-CD38+ (%)
**G.E.#1**	88.6	99.3	16
**B.I. #2**	89.8	96.98	4.68
**S.G.#3**	89.5	98.45	1.25
**S.D.#4**	66.3	94.9	10.8

Figure 2 Suppl

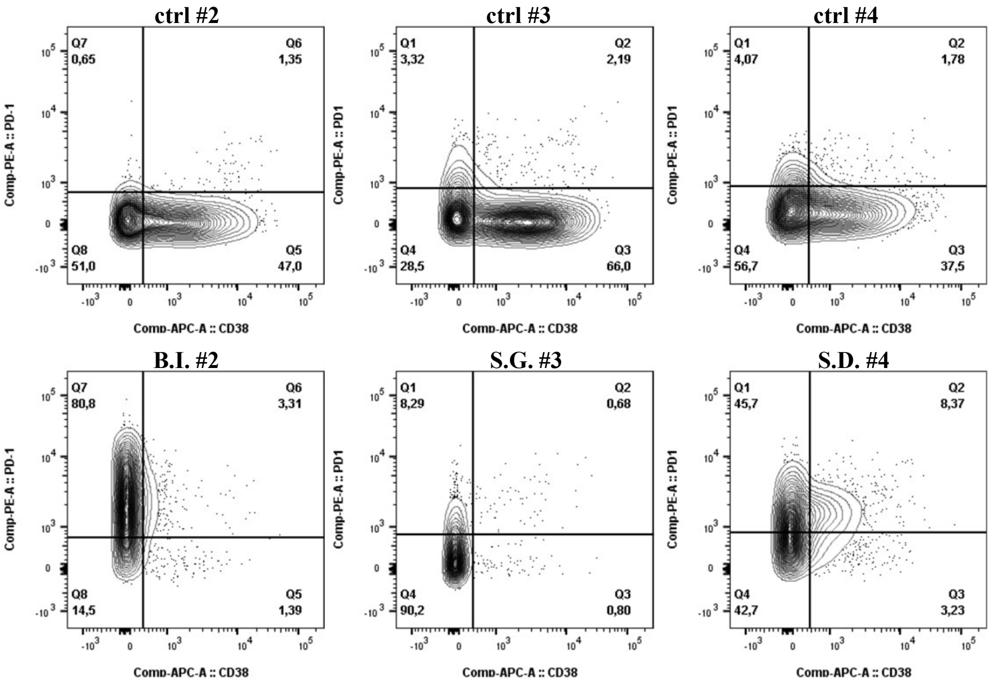

Healthy ctrl	CD4+CD38+(%)	CD4+PD-1+ (%)	Patients	CD4+CD38+(%)	CD4+PD-1+ (%)
#2	48.35	2	B.I. #2	4.7	84.11
#3	68.19	5.51	S.G.#3	1.48	8.97
#4	39.28	5.85	S.D.#4	11.6	54.07

Figure 3 Suppl
