# Supplementary material for: CD38 Expression by Circulating and Skin-Infiltrating Lymphocytes from Sezary Syndrome Patients: A Flow Cytometry and Immunohistochemistry Study
Source: Dis Markers. 2022 Feb 24;2022:3424413. doi: 10.1155/2022/3424413 (PMC8896155; doi:10.1155/2022/3424413)
Supplement: Supplementary 2 — Table 1 Supplementary: clinical characteristics of CD38+ and CD38- patients. [file 3424413.f2.docx]

**Table 1 Supplementary**. Clinical characteristics of CD38+ and CD38- patients

|  | CD38+ * | CD38- |
| --- | --- | --- |
| Total | 12 | 64 |
|  |  |  |
| Male/female | 5/7 | 34/30 |
| Age at diagnosis  (median; range) | 59 years (50-102) | 66 years (33-95) |
| CD4/CD8 ratio  (median; range) | 19.5 (2-98) | 14.5 (1.5-98) |
| Circulating Sézary cells** (mm3/; median; range) | 4,789 (1,000 – 31,127) | 2,361 (1,000-54,420) |
| Overall survival  (median; range) | 22.2 months  (1.6 months-9 years) | 25.2 months  (2.9 months – 12 years) |

* Patients were considered CD38 positive if they have blood circulating lymphocytes and/or skin infiltrates positive for CD38 expression.

** Absolute number of circulating Sézary cells defined on the basis of flow-cytometry data considering CD26, presence of restricted Vbeta expression, “dim” expression of T-cell lineage markers or other atypical phenotype

Statistical test used: for age and gender: chi-square test; for the number of circulating Sézary cells: the Student T test; for survival the Kaplan Meier method and log-rank test. Differences were not statistically significant
